# Supplementary figures and images for: Impeding pathways of intrinsic resistance in Escherichia coli confers antibiotic sensitization and resistance proofing
Source: PLoS Biol. 2025 Oct 22;23(10):e3003443. doi: 10.1371/journal.pbio.3003443 (PMC12543169; doi:10.1371/journal.pbio.3003443)

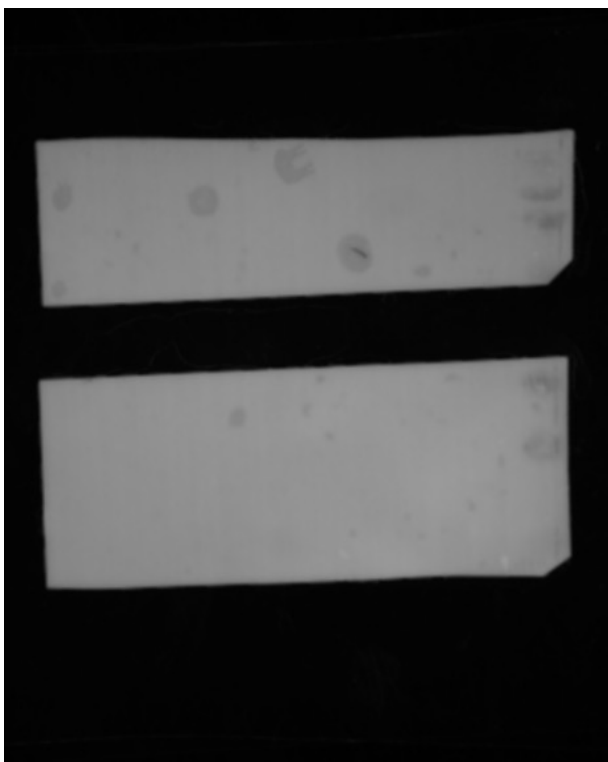

Epi-white

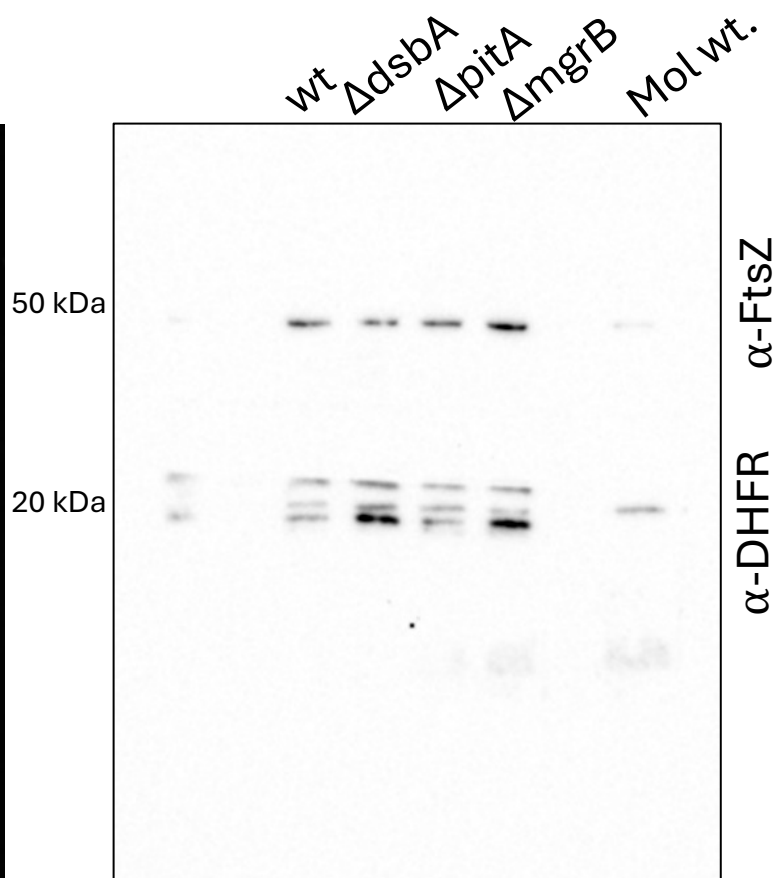

Chemiluminescence

Supplement: S1 Text — (PDF) [file pbio.3003443.s004.pdf]
