# Supplementary material for: Impeding pathways of intrinsic resistance in Escherichia coli confers antibiotic sensitization and resistance proofing
Source: PLoS Biol. 2025 Oct 22;23(10):e3003443. doi: 10.1371/journal.pbio.3003443 (PMC12543169; doi:10.1371/journal.pbio.3003443)
Supplement: Attachment — Submitted filename: Response to reviewers comments.docx [file pbio.3003443.s007.docx]

Reviewer #1:

The aim of this experimental study is to assess the potential of limiting antibiotic resistance emergence by reducing the intrinsic resistance of bacteria. Using E. coli as a model bacterium, the authors identify central genes in the intrinsic resistance to chloramphenicol and trimethoprim (lpxM, rfaG, acrB). They then demonstrate that deletion (as a proxy for inhibition) of these genes indeed increases sensitivity and reduces the ability to evolve resistance to regular doses of trimethoprim. However, at reduced doses of trimethoprim, resistance emerges by adaptive evolution in all three cases. The acrB efflux mutant appears to be more limited compared to the others and thus the authors test the ability of chemical inhibitors of efflux to similarly suppress resistance emergence. Importantly they find that bacterial adaptation rapidly overcomes the chemical inhibitor, identifying a significant limitation of this approach for treatment.
There is little one can criticize in the conducted work. The authors clearly know what they are talking about and all experiments include relevant controls and analyses. The paper provides enough details of its methodology so that its experiments could be reproduced. The results are well presented and the manuscript is well written (clear and accessible to a wide audience). The main claims are:
1) There is a significant overlap in the intrinsic resistance to trimethoprim and chloramphenicol, in which lpxM, rfaG and acrB are central players.
2) Reduction of intrinsic resistance by deletion or inhibition reduces resistance emergence
3) Inhibition of intrinsic resistance is rapidly overcome by evolution.
Generally, these points are each well supported by the presented data. The findings would be a significant contribution and suitable for publication with PLOS Biology. However, one aspect of claim 2 may be strengthened (although this is not required; major point 1). I also have some concerns about fair treatment of the literature (major point 2).

Response: We appreciate the generally positive comments from the Reviewer, as well as the succinct summary of our main findings. We have made several revisions, including adding new data as suggested by the Reviewer which, in our opinion, address the main concerns highlighted here. Point by point explanations are provided below.

Major points
MP1.
One aspect is that the rescue experiments (Fig 4a) are done at MIC of the wild type. As the three mutants are hypersensitive, the used dosage is effectively 4xMIC (acrB, lpxM) or 2xMIC (rfaG). What is not fully clear is whether the observed extinction is caused by genetic limitation of the mutant (inaccessibility of genetic pathways), or whether the rescaling of the susceptibility is what causes the constraint (greater step required). A control experiment here would be to test the ability of the WT to adapt to 2xMIC and 4xMIC. As only 3/6 WT lineages survived the MIC challenge, a high number of replicate cultures would be required to achieve the required resolution with this analysis.

Response: The Reviewer brings up an interesting idea here. The two models outlined in this comment are both likely, though we now add some additional data to the manuscript that differentiates between them. To address this comment, we have, firstly, repeated our short term evolution experiments (i.e. for 5 growth cycle) by increasing the replicate number to 30 for each genotype-drug combination. Secondly, for wild type bacteria, we have also included evolution at 2x and 4xMIC of trimethoprim as suggested. Indeed, we find that increasing the number of replicates has provided greater resolution, as hence greater insight into the question of potential for resistance evolution of hypersensitive strains. In light of these new data (which are added to Figure 4 and 7) we are able to make more robust claims in the revised manuscript. These are listed below:

i. At MIC, the number of surviving replicates of *lpxM* and *rfaG* knockouts are marginally lower than wild type. However, the *acrB* knockout shows a substantially more dramatic reduction in survival. At MIC/9, all replicates of wild type, *lpxM* and *rfaG* knockouts survive till the 5^th^ serial transfer. On the other hand, the *acrB* knockout shows some lethality even at this low drug exposure (Figure 4B). Further, the OD values of cultures at Day 5 of the evolution show that the *acrB* knockout has severely compromised adaptability, i.e. surviving populations tend to have lower ODs than wild type populations cultured for a similar duration at MIC/9 trimethoprim (Figure 4C). These new data demonstrate that the *acrB* knockout has substantially lower potential for adaptation to the antibiotic than the other two hypersensitive strains at high as well as low drug pressures. Based on these new data, we cement our original claim that inhibiting efflux may be a more effective resistance proofing strategy than cell wall biosynthesis.

ii. Carrying capacities of the different hypersensitive mutants across antibiotic concentrations are lower than wild type, but not substantially different from one another (Figure 4A). Yet, their adaptability to trimethoprim is dramatically different, i.e., knockout of *acrB* is severely compromised compared to *lpxM* and *rfaG*. Thus, their different adaptabilities are unlikely to be due to differences in population size and mutation supply. Instead, we show that similar adaptive mutations provide smaller benefit in the *acrB* knockout than in other mutant backgrounds. These new data, taken together with older data showing greater reduction in drug IC50 in trimethoprim resistant strains by loss of *acrB* than *rfaG* (Figure 6E), more convincingly provide a mechanistic explanation for the differences between efflux and cell wall biosynthesis mutants.

iii. Chlorpromazine+Trimethoprim (CPZ+TMP) is more detrimental than the *acrB* knockout in terms of number of surviving populations at low drug (Figure 7B). This insight was only revealed once we increased the number of replicates. However, surviving lineages of CPZ+TMP evolve higher resistance than surviving lineages of *acrB* knockout. This nuance is explained by our earlier data regarding the evolution of *acrR* mutations in the CPZ+TMP lines (Figure 8).

MP2.
The authors somewhat neglect a large body of work on resistance evolvability. Here, there is considerable recent work (e.g. <https://pubmed.ncbi.nlm.nih.gov/39984459/> <https://pubmed.ncbi.nlm.nih.gov/32561723/> ). I am especially concerned that the authors do not relate their work to two previous pieces of Guillaume Cheverau and Tobias Bollenbach where the Keio library was used to identify genetic constraints on evolvability, importantly including chloramphenicol, trimethoprim and their combination (<https://pubmed.ncbi.nlm.nih.gov/26581035/> <https://pubmed.ncbi.nlm.nih.gov/25924924/> ). Within these previous studies genes lpxM and rfaG are indeed already identified and associated to the efficacy of chloramphenicol and trimethoprim. I would strongly encourage the authors to relate their findings to this previous work.

Response: We appreciate that this was an oversight that could have been avoided. We have now added the suggested citations in the revised introduction and discussion sections of our manuscript.

Minor points:
L. 55. What are LMICs? Please avoid unexplained abbreviations

Response: Full form provided in the revised manuscript.

L. 80. No references provided for this statement. Some would say that the drug discovery pipeline has been accelerating in the last 10 years.

Response: We have added a reference, and softened our claim as the Reviewer does make a valid point here regarding revival in antibiotic discovery pipelines.

L. 86. Please rationalize, why chloramphenicol and trimethoprim were chosen as antibiotics of study

Response: These antibiotics are chemically diverse, target different molecular pathways, have non-overlapping resistance mechanisms and are both broad spectrum bacteriostatic antibiotics. We have provided this justification in the revised manuscript.

L. 123 ff. I understand why the authors chose not to continue the work with the mutants that had generally impaired growth, however, from a treatment perspective, wouldn't these be the most promising candidates?

Response: Indeed, the reviewer is correct in their observation here. However, mutants with growth defects are generally also more likely to evolve suppressors and compensatory mutations. This, we expected, to result in unstable hypersensitivity. We therefore worked with knockouts that were not intrinsically slow growing. We would like to add, though, that this manuscript is the first of an ongoing project aimed at looking at how hypersensitive mutants recover under drug pressure. We plan to include a broader set of mutants and antibiotics going ahead, including some slow-growing mutants. Hopefully, these ongoing experiments will throw light on how the growth defects associated with antibiotic hypersensitivity affect the resistance proofing properties of such mutants.

------------------------------------


Reviewer #2 (Markus Seeger):

This nicely presented paper of the Matange lab describes a large set of experiments geared towards understanding the involvement of the drug efflux pump AcrB (among other intrinsic resistance mechanisms) to influence/drive the evolution of drug resistant strains. The study focussed on the two antibiotics trimethoprim and chloramphenicol. The E. coli K-12 lab strain served as model system. In a first set of the study, the authors screened the Keio collection for gene deletions that render E. coli hypersensitive towards trimethoprim or chloramphenicol, thereby assessing the "intrinsic resistome" of E. coli towards these two drugs. Thereby, the authors found around 20 genes, and then focussed mainly on drug efflux pump acrB, the lipopolysaccharide glucosyl transferase I rfaG and  Lipid A myristoyl transferase lpxM. Using clean E. coli ΔacrB, ΔrfaG, ΔlpxM strains, the authors then addressed the question how trimethoprim and chloramphenicol treatment drive the evolution of drug
resistance in these strains versus the isogenic wt E. coli strain at different drug concentrations. While at a drug concentration of 1xMIC of wt E. coli, not surprisingly none of the mutant strains survived. Nevertheless, the experiments showed that if one can efficiently interfere with these intrinsic resistance pathways, one may achieve desirable "drug-proofing" effects, meaning that one can potentially avoid or at least reduce the emergence of novel mutations that confer drug resistance.
Interestingly, at sub-MIC levels the evolutionary trajectories varied greatly. In the course of these experiments, the authors uncovered novel factors contributing to trimethoprim resistance (i.e. DsbA and PitA). They worked out the mechanisms, i.e. that genetic deletion of dsbA and pitA results in higher expression level of the trimethoprim target DHFR.
In a final set of experiments, the authors compared the evolutionary trajectory of the genetic deletion mutant of acrB versus a pharmacological intervention of drug efflux by including drug efflux inhibitor (namely chlorpromazine). Very interestingly, the authors found that in the presence of chlorpromazine, the most frequent evolutionary consequence was deletion of the acrAB operon repressor acrR, thereby leading to constitutive overproduction of AcrAB and consequently cross-resistance towards a larger set of drugs which are AcrB substrates.
Overall, this is a creative paper that is very nicely written and interesting to read. It contains a large amount of high quality data, which are the result of intense and focussed research, likely over many years. I find many of the interpretations as highly relevant for the field of drug efflux, and the study elaborately leads through the labyrinth of co-acting resistance mechanisms and epistasis trajectories. As a somewhat inevitable side effect, the complexity of the data and their interpretation is rather high; hence it is not an easy read.
The comments below are mostly suggestions how the story may be (further) streamlined to bring the main messages across in a clearer fashion.

Response: We thank the Reviewer for his positive comments on our manuscript. We have addressed most of his suggestions and provide a point by point response below.

Main points
1) I consider the authors' finding of acrR mutations as a result of efflux inhibitor use at sub-MIC concentration as the most striking finding with the highest impact on the drug efflux field. Although the EPI is mentioned in the abstract, the authors can do a better job in saying that there is a fundamental difference in genetically deleting acrB versus inhibiting it pharmacologically with an EPI in terms of evolutionary outcome and that the use of EPIs may lead to undesirable collateral damage, at least in this specific case. Maybe, the authors find a way to stress this already in the title instead of mentioning the "resistance proofing" there, which in my view is less interesting/relevant in the context of this study. Having said all this, it is entirely up to the authors to follow this suggestion.

Response: We agree that the difference we report between genetic and pharmacological inhibition of AcrB are an important finding. Yet, since this only represents the final result of the paper, we are not inclined to change the title of the paper. We have, however, added a line to emphasise this result in the abstract. We hope that this revision satisfactorily emphasises this key finding from our study.

2) The paper claims in the introduction (line 84) that "targeting intrinsic resistance mechanisms as resistance proofing strategies, i.e. preventing de novo resistance evolution, remains to be explored".  While I agree that comparatively little is known, a quick literature search using the terms "evolution drug efflux inhibiton" revealed quite a number of interesting, recent papers: PMID: 36400010, PMID: 32769975, PMID: 39982069. These three in my view relevant papers have not been cited. With a somewhat deeper search, I am sure the authors will find another few good papers in the realm of drug efflux and evolution. I suggest the authors extend their literature search and discuss their findings in light of the newest literature. By no means, these papers undercut the novelty of the authors' work; rather to the contrary, they illustrate that investigation of drug efflux pumps in the context of antimicrobial resistance is a "hot" science topic with a good momentum.

Response: This is indeed an oversight on our part. We have added the references that the Reviewer highlighted, along with a few others related to the impact of efflux and resistance evolution, in the introduction and discussion sections of the revised manuscript.

Minor points
1) Line 111 and Fig. 1B: it was interesting to see that some gene deletions of the Keio collection also conferred stronger growth in the presence of antibiotics. What is going on with these?

Response: Indeed, mutants from the Keio collection that grew better in the two antibiotics are of great interest. This is because they likely represent novel pathways of resistance. Since this study was restricted to hypersensitive mutants, we have not highlighted the resistant knockouts in this manuscript. However, we are currently characterising some of them in our lab. One of the top hits from the screen was *mgrB*, that we have already shown to be a spontaneous mutant that drives trimethoprim resistance. Another hit, *rng*, also seems to confer substantial benefit in trimethoprim, but has not been reported so far. The mechanism of resistance of the *rng* knockout appears to be independent of known trimethoprim resistance pathways and will hopefully be deciphered by some of the ongoing experiments in our lab.

Notably, we do not find too many common resistant knockouts between the two antibiotic screens. This seems to suggest that while there are several common mechanisms of intrinsic resistance, adaptive resistance likely relies mostly on drug specific mechanisms.

2) Line 115: mention here what kind of pipeline/method was used to group genes into functional clusters. It is surely mentioned in the methods, but I would quickly state it here as well.

Response: We use the Ecocyc database for these annotation. This information has been added to the revised manuscript.

3) Line 130: I was very surprised the authors did not pick up the other components of the AcrAB-TolC system in their Keio screen: acrA and tolC. Any explanation for this?

Response: Indeed, this is an insightful comment and we thank the Reviewer for bringing it up. Our cut-offs for classifying mutants as hypersensitive was very stringent, i.e. at least two standard deviations from the median. Further, we excluded any hits that gave us a large variation between replicates. As a result, *acrA* did not make the cut in the trimethoprim screen, while *tolC* did not make the cut in the chloramphenicol screen. *AcrB* was the only mutant that made the short list across both antibiotics, which is why we took it forward. However, we have since generated the single knockouts of all three genes and confirmed that all three are hypersensitive to multiple antibiotics. These data are part of ongoing studies in the lab and will hopefully form the part of a future manuscript.

4) Line 137: it would be of tremendous help for the reader to extend the description of rfaG and lpxM here. Pls consider adding a supplementary Figure drawing the respective pathways and explain in more detail about the physiology of these pathways and how they are potentially linked to trimethroprim and chloramphenicol resistance. It may also not harm to include a drawing of the AcrAB-TolC system in such an additional Supplementary Figure.

Response: We appreciate this suggestion. To clarify this point we have added a brief description of how we think these mutants many be resulting in broad hypersensitivity to antibiotics. In all three mutants, we expect that there is greater intracellular antibiotic concentration achieved than the wild type. While *acrB* deletion does this by preventing efflux, *lpxM* and *rfaG* deletion do this by greater permeation into the cell due to cell envelope perturbation. However, we have not represented this with a figure, since the above description is only a prediction based on the known functions of these genes. We do not have hard data demonstrating this and hence refrain from making a more definitive claim.

5) Line 141: the authors re-introduced the gene deletions into the MG1655 strain. Why didn't the authors use the Keio strains right away?

Response: We have found that, occasionally, mutants from the Keio collection (i.e. the original knockouts in *E. coli* BW25113 background) have secondary mutations (possibly due to repeated sub-culturing) that may alter phenotypes. Further, on very rare occasions we also find that some Keio knockouts are mislabelled. Since we wanted to take forward the three gene knockouts (*acrB*, *lpxM* and *rfaG*) for more focussed study including evolution experiments, we moved them into a clean MG1655 background and confirmed these with PCRs to negate any possible artefacts.

6) Line 169: suggested to cite papers wherein the term "resistance proofing" has been coined/first used

Response: We thank the Reviewer for this comment. Unfortunately, we were unable to locate the very first instance of usage of this term in literature. However, we have added a few references that describe this term to provide greater context.

7) Line 177 and line 192: I do not consider it as surprising that these deletion strains stopped growing considering that the MIC of trimethroprim was several fold above their MIC (= 1xMIC for wt E. coli).

Response: In light of the new data that we have added to Figure 4 and 7, we think that merely a lower MIC does not guarantee resistance proofing. As the Reviewer will see, knockouts of *acrB*, *lpxM* and *rfaG* were similarly compromised in trimethoprim (Fig. 4A). Yet they showed markedly different propensities for resistance evolution. We have attributed this difference to the interaction between these specific perturbation of intrinsic resistance pathways and adaptive mutations. It appears, therefore, that understanding the potential for resistance evolution is more complex than it appears to be at face value.

8) Line 251: Consider giving a new section title here. The entire section is quite long.

Response: We thank the Reviewer for this suggestion. However, since this section is thematically coherent, we have retained it as a single (and admittedly rather long!) section.

9) Line 281: Citation 19 does not show a structure of AcrB with chloropramizine, but only a docking result. That's not a proof for specific binding. I would assume that chloropramizine quite broadly inhibits all kinds of pumps. You may also have a brief look at this review on drug efflux inhibition in TB: PMID: 34637511. But quite clearly, chloropramizine also appears to block AcrB function; hence in terms of interpretation of results, all is fine.

Response: We thank the Reviewer for pointing out this mistake. We have re-worded this section to reflect that chlorpromazine (CPZ) may be binding directly to AcrB. We have also added the recommended reference to the revised manuscript. Importantly, new data in Figure 7 shows that the effects of chlorpromazine are more dramatic than *acrB* deletion, in terms of the numbers of surviving replicates at MIC/9 trimethoprim. This observation reinforces the point made by the Reviewer that CPZ may not be specific to AcrAB, and we have mentioned this possibility in the revised manuscript.

10) Line 328: I consider this finding as the core finding of the paper. Pls also include that more clearly into title/abstract such the message is really seen.

Response: We have emphasised this in the abstract, but have chosen to not alter the title.

11) Line 398-401: I cannot follow the logics of the argument. Pls try to express yourself clearer.

Response: We have re-written this line to provide more clarity.

12) Fig. 1C: I would list the "hypotheticals" last.

Response: We have reorganised the categories in the legend to be in line with the graph. At the moment “Hypotheticals” remains at the top of the list, but we can move it down if the Reviewer/Editor feel that it substantially distracts from the main point being made.

13) Fig. 7A: The Checkerboard results look a bit odd/non-uniform. Has the experiment been repeated several times? Nevertheless, it is not critical for the evolution experiments performed with chloropramizine.

Response: Indeed, the OD values plotted in figure 7A are averaged across 3 replicates. We do agree that the data look odd, specifically in the region of high TMP and low CPZ. However, when we analysed the individual replicates, we realised that this drug-combination regime gave us the most variation as well. We are unsure of why this has happened, but as the Reviewer points out, it does not substantially alter our claims. We have, therefore, not altered the figure.

------------------------------------


Reviewer #3:

Balachandran et al. began this study with a screen of the well-characterized Keio single gene deletion collection to identify isolates resistant to trimethoprim or chloramphenicol. Of the deletions that conferred resistance, they chose to focus on acrB, the very well-studied component of the essential AcrAB-TolC multidrug efflux pump, as well as rfaG, lpxM, and nudB (previously known to confer susceptibility to trimethoprim). They introduced these deletions into an isogenic background, E. coli K-12 MG1655, and documented varying degrees of susceptibility to TMP-SXT, chloramphenicol and others.

Next, they deleted acrB, lpxM, and rfaG from laboratory evolved resistant isolates from a prior study, and measured susceptibilities to a few different antibiotics.  They document that acrB deletion confers susceptibility to a variety of antibiotics, consistent with a number of prior studies on acrAB-TolC, and suggesting that the AcrAB-TolC complex likely played a role in conferring resistance in these laboratory evolved isolates, again consistent with prior studies. The authors then challenge these engineered strains with serial passage through high concentrations of trimethoprim to evolve resistance. Here they find (perhaps not surprisingly) that isolates with deleted acrB, lpxM, and rfaG remained susceptible and/or were driven to extinction during the passage experiments at high trimethoprim concentrations that likely substantially inhibited replication (and therefore evolvability).

The authors repeated the experiments at sub-MIC concentrations of antibiotics at which replication would be expected to be permitted, and in these experiments they observed the evolution of resistant (or less susceptible) lineages. They then characterized these lineages by sequencing, and found mutations in some expected targets, including DHFR, the target of trimethoprim, as previously studied by others.  They identify a few other targets and interpret their relevance with additional knock out experiments and reasoning based solidly on what is known in the literature.  Finally, they performed adaptive evolution experiments with chlorpromazine to inhibit the ArcAB-TolC pump, and found that disruptions in AcrR (the repressor of the acrAB operon) were selected, as expected and consistent with what is known from other studies.

The authors clearly did a lot of careful work for this study, and the manuscript is written in a way in which the experiments and results can be followed throughout. Additionally, the figures are well organized, easy to follow, and information-rich.  I had trouble, however, with understanding what exactly the novel or reportable conclusions were, and I found that the qualitative approach to the analysis of the adaptive evolution experiments made quantitative interpretation difficult.  Specific comments follow.

Response: We thank the Reviewer for this summary of our manuscript. We appreciate their concerns, which we have addressed through several modifications including new data, in the revised manuscript. Point by point responses are provided below.

Novelty:  I think the authors should try to do a more convincing job of explaining exactly what is/are the novel, reportable findings in this work and how the specific novel findings advance our understanding of resistance and evolvability beyond what is known from the >1000 papers published in this area.  For instance, the AcrAB-TolC has been extensively studied, and its role in both natural and evolved resistance is well documented and well-understood (as are it's homologs MexAB-OprM, etc).  Likewise, many of the other gene targets that were studied are well characterized (for instance acrR, rfaG, lpxM, folA, etc), and the findings the authors report are generally consistent with published literature, or what would be expected, based on what is known.  The concept of "resistance proofing" is interesting, but, for instance, the demonstration that isolates with deleted AcrB go to extinction at high concentrations of trimethoprim is not itself a demonstration of a new
phenomenon, given the well-understood role that AcrAB-TolC plays in mediating resistance to a variety of chemically-dissimilar antibiotics.  If there is something novel that is being presented to support this concept, I think the authors should try to do a better job to explain exactly what the new finding is (beyond the large and very well-organized mutational dataset that is presented).

Response: We appreciate the concern raised by Reviewer 3. This is indeed a busy area and we have modified the discussion section of our revised manuscript to highlight more clearly the novel results and analyses we present here. Briefly, these are as follows:

i. While it is well established that efflux and cell wall biosynthesis are two of the primary pathways resulting in intrinsic antibiotic resistance, our study address which of these are more likely to also result in resistance proofing. We show that efflux is indeed a superior pathway than cell wall for resistance proofing, even though perturbing either of these can be used for antibiotic sensitisation.

ii. A major novel aspect of our study, as the Reviewer themselves identify, is the idea of resistance proofing. While related to the more frequently explored “evolvability” concept, resistance proofing is by definition related to the ability of a bacterial population to adapt to an extent that allows for growth above a certain cut-off concentration. In a clinical context, this would be the MIC. Several evolvability studies use a morbidostat, i.e. dynamically changing concentrations of antibiotic, to estimate the potential for evolution. However, our study shows that the potential for adaptation does not necessarily translate to crossing of clinically relevant breakpoints.

iii. Several studies, some of which were also pointed out by Reviewer 1, choose antibiotics like tetracycline, chloramphenicol or ciprofloxacin. Bacteria like *E. coli* directly engage efflux systems to evolve resistance against these antibiotics. This biases the outcomes of evolution experiments with efflux deficient mutants. However, our study shows that even when adaptive mutations are not in efflux pathways, loss of the major AcrAB-TolC system can fundamentally alter the potential for resistance evolution.

iv. The lack of concordance between genetic and pharmacological inhibition of efflux is a major point that is made by the current study, as highlighted also by Reviewer 2. This will hopefully have an impact on the area of EPI research and steer the discovery of EPIs towards those that are less affected by expression level changes, as we have argued in the discussion of our manuscript.

We are in the process of exploring these effects with a larger number of antibiotics and hypersensitive mutants. Hopefully we will be able to test more robustly the generalisability of our claims in the future. Yet, we believe that current manuscript has enough new and topical messages that it should be published now, to be followed up by data on additional mutants and antibiotics in a subsequent paper.

Quatntiative rigor:  The "evolvability" findings at high and low trimethoprim MICs (presumably corresponding to low and high permitted replication rates) are generally consistent with what is well-understood concerning the relationships between replication rates, critical population size, the pool of mutations available for selection, and the rate at which mutations contributing to resistance are discovered by the population. The experiments themselves seem well done, but the analysis has been treated in much more quantitative detail by others over the past decades.  If the authors want to make a new argument about selection in these regimes of high and low replication rates, then at the very least the argument will need to be formulated rigorously and quantitatively with actual calculation of generation times at high and low antibiotic concentrations, critical population numbers, and the estimation of mutational pool given the mutation rate per generation.  I could not find
the starting cell density/exact cell count in the methods. Reference 30 is given, but this critical variable and needs to be included explicitly (the authors will find that the results of their evolvability experiments indeed likely depend critically on the starting cell density, particularly in the experiments with high trimethoprim concentration).

Response: We thank the Reviewer for pointing out this very valid concern. We have now added several new pieces of data and also provided greater quantitative details regarding population sizes at different TMP concentrations that have enhanced the robustness of our conclusions (Figures 4 and 7). As outlined in the revised manuscript, based on the population sizes of mutants and wild type at different TMP concentrations, as well as known mutant frequencies in *E. coli*, the differences in potential for resistance evolution between hypersensitive strains appears to not stem from different mutation supply (i.e. different population sizes). Instead the *acrB* knockout seems to evolve resistance less effectively as adaptive mutations produce milder benefit in this background. Once again, this is particularly interesting for trimethoprim as resistance to trimethoprim does not directly involve mutations in efflux pathways. We hope that these additional data and a more quantitatively written description of our results provide more rigour and improve confidence in our results.

Genome sequencing:  Why were the evolved populations expanded in antibiotic free media prior to sequencing?  Mutations conferring resistance with high fitness costs may not be represented in the post-expansion population (this has been demonstrated by others), and additionally the proportions of mutations present would not be expected to reflect the original proportions under selection.  What are the authors' thoughts on this?

Response: This is indeed a valid concern. We revive our frozen stocks in drug free media routinely as this ensures that all cultures, regardless of the MIC they have evolved give similar yields of DNA for genome sequencing. As the Reviewer points out, this may result in the loss of rare mutations that are costly. However, we do not think this is a major concern in the case of our analyses for the following reasons. Firstly, we are only interested in the major pathways of adaptation that occur reproducibly across replicates, and since we have sequenced populations under antibiotic pressure for 20 cycles of growth (~140 generations) we expect the adaptive mutations to be enriched sufficiently by this point of time. Indeed this is why we impose a frequency cut-off of 20% for all our analyses. Secondly, it is expected that at low antibiotic pressure, unlike high antibiotic pressure, costly mutations are unlikely to get enriched in evolving bacterial populations. Finally, we have also tested for population IC50 values for a few representative lineages when revived in drug-free and TMP-supplemented media, and we do not find significant differences. Thus, we don’t expect major qualitative differences in mutational landscapes due to revival in drug-free media.
