# Supplementary material for: Impeding pathways of intrinsic resistance in Escherichia coli confers antibiotic sensitization and resistance proofing
Source: PLoS Biol. 2025 Oct 22;23(10):e3003443. doi: 10.1371/journal.pbio.3003443 (PMC12543169; doi:10.1371/journal.pbio.3003443)
Supplement: Attachment — Submitted filename: Response to comments.docx [file pbio.3003443.s008.docx]

**Response to editor/reviewer comments**

Based on the reviews, we are likely to accept this manuscript for publication, provided you satisfactorily address the remaining editorial points. Please also make sure to address the following data and other policy-related requests.

a) We routinely suggest changes to titles to ensure maximum accessibility for a broad, non-specialist readership, and to ensure they reflect the contents of the paper. In this case, we would suggest an edit to the title, as follows. Please ensure you change both the manuscript file and the online submission system, as they need to match for final acceptance:

"Impeding pathways of intrinsic resistance in Escherichia coli confers antibiotic sensitisation and resistance proofing"

Response: Done.

b) Please add the link to the funding agencies in the Financial Disclosure statement in the manuscript details.

Response: Done.

c) You may be aware of the PLOS Data Policy, which requires that all data be made available without restriction: <http://journals.plos.org/plosbiology/s/data-availability>. For more information, please also see this editorial: <http://dx.doi.org/10.1371/journal.pbio.1001797>
Please supply the numerical values either in the a supplementary file or as a permanent DOI’d deposition for the following figures:
Figure 1BC, 2, 3ABC, 4ABCD, 5, 6ABDEF, 7BC, 8AB
NOTE: the numerical data provided should include all replicates AND the way in which the plotted mean and errors were derived (it should not present only the mean/average values).

→ It is not clear if the Supplementary File 1 contains this data. If this is the case, please separate them according to the figure for easier access

Response: Following are a list of data that have been added, or clarifications in response to this comment.

Figure 1B and C: The raw data have been added to Supplementary File 1. Tabs in the spreadsheet have been annotated accordingly.

Figure 2: Raw data have been added to Supplementary File 2. Tabs in the spreadsheet have been annotated accordingly.

Figure 3A, B, C: Raw data have been added to Supplementary File 2. Tabs in the spreadsheet have been annotated accordingly.

Figure 3 B,C: Individual measurements are now plotted.

Figure 4 A,D: Raw data have been added to Supplementary File 2. Tabs in the spreadsheet have been annotated accordingly.

Figure 4B: Raw numbers from a single experiment are directly plotted in this graph. Also the raw numbers are mentioned in the running text.

Figure 4C: Raw numbers from a single measurement are directly plotted in this graph.

Figure 5: Raw data are now provided in Supplementary File 3.

Figure 6A,F: Raw data have been added to Supplementary File 2. Tabs in the spreadsheet have been annotated accordingly.

Figure 6B,D: Individual measurements are now shown in the respective graphs.

Figure 6E: The data points are the means from 6D, for which individual replicates are now included.

Figure 7B: Raw numbers from a single measurement are directly plotted in this graph.

Figure 7C: Raw data have been added to Supplementary File 2. Tabs in the spreadsheet have been annotated accordingly.

Figure 8A: Raw data have been added to Supplementary File 3.

Figure 8B: Raw data have been added to Supplementary File 2. Tabs in the spreadsheet have been annotated accordingly.

d) Please cite the location of the data clearly in all relevant main and supplementary Figure legends, e.g. “The data underlying this Figure can be found in S1 Data” or “The data underlying this Figure can be found in <https://doi.org/10.5281/zenodo.XXXXX>”

Response: Done.

e) Please ensure that you are using best practice for statistical reporting and data presentation. These are our guidelines <https://journals.plos.org/plosbiology/s/best-practices-in-research-reporting#loc-statistical-reporting>  and a useful resource  on data presentation <https://journals.plos.org/plosbiology/article?id=10.1371/journal.pbio.1002128>

→ If you are reporting experiments where n ≤ 5, please plot each individual data point.

Response: We have added individual data points wherever needed.

f) Please make sure that all figures use a colorblind-friendly palette.

Response: Done.

g) Per journal policy, if you have generated any custom code during the course of this investigation, please make it available without restrictions. Please ensure that the code is sufficiently well documented and reusable, and that your Data Statement in the Editorial Manager submission system accurately describes where your code can be found.

-- Please note that we cannot accept sole deposition of code in GitHub, as this could be changed after publication. However, you can archive this version of your publicly available GitHub code to Zenodo. Once you do this, it will generate a DOI number, which you will need to provide in the Data Accessibility Statement (you are welcome to also provide the GitHub access information). See the process for doing this here: <https://docs.github.com/en/repositories/archiving-a-github-repository/referencing-and-citing-content>

Response: Not applicable to our study.

As you address these items, please take this last chance to review your reference list to ensure that it is complete and correct. If you have cited papers that have been retracted, please include the rationale for doing so in the manuscript text, or remove these references and replace them with relevant current references. Any changes to the reference list should be mentioned in the cover letter that accompanies your revised manuscript.

Response: No change needed, to the best of our knowledge.

------------------------------------------------------------------------
REVIEWERS' COMMENTS
------------------------------------------------------------------------


Reviewer #1:

I have read the author replies and revisions and can confirm that the new data and changes to the test satisfactorily addressed my concerns. I can now recommend publication of the paper.

------------------------------------------------------------------------


Reviewer #2 (Markus Seeger):

The authors have adequately addressed all my concerns and I consider the paper to be ready to be published in this form.

------------------------------------------------------------------------


Reviewer #3:

The authors have extensively revised the manuscript and it is substantially improved.  My specific concerns have been sufficiently addressed, and I commend the authors for their excellent work in this interesting study.

Response: We thanks the Reviewers for their appreciation of our revised manuscript.
